# Underground Parking Lot Navigation System Using Long-Term Evolution Signal

**DOI:** 10.3390/s21051725

**Published:** 2021-03-02

**Authors:** Beomju Shin, Jung Ho Lee, Changsu Yu, Chulki Kim, Taikjin Lee

**Affiliations:** Sensor System Research Center, Korea Institute of Science and Technology, 5, Hwarang-ro 14-gil, Seongbuk-gu, Seoul 02972, Korea; bjshin@kist.re.kr (B.S.); 213010@kist.re.kr (J.H.L.); cs.yu@kist.re.kr (C.Y.); chulki.kim@kist.re.kr (C.K.)

**Keywords:** underground parking lot navigation, smartphone, LTE

## Abstract

Some of the shopping malls, airports, hospitals, etc. have underground parking lots where hundreds of vehicles can be parked. However, first-time visitors find it difficult to determine their current location and need to keep moving the vehicle to find an empty parking space. Moreover, they need to remember the parked location, and find a nearby staircase or elevator to move toward the destination. In such a situation, if the user location can be estimated, a new navigation system can be offered, which can assist users. This study presents an underground parking lot navigation system using long-term evolution (LTE) signals. As the proposed system utilizes LTE network signals for which the infrastructure is already installed, no additional infrastructure is required. To estimate the location of the vehicle, the signal strength of the LTE signal is accumulated, and the location of the vehicle is estimated by comparing it with the previously stored database of the LTE received signal strength (RSS). In addition, the acceleration and gyroscope sensors of a smartphone are used to improve the vehicle position estimation performance. The effectiveness of the proposed system is verified by conducting an experiment in a large shopping-mall underground parking lot where approximately 500 vehicles can be parked. From the results of the experiment, an error of less than an average of 10 m was obtained, which shows that seamless navigation is possible using the proposed system even in an environment where GNSS does not function.

## 1. Introduction

Seamless positioning is one of critical factors in future navigation systems. Currently, vehicle navigation systems provide guidance only around buildings, and the navigation service is terminated on entering underground parking lots. However, in large underground parking lots such as in hospitals, shopping malls, and airports, it is difficult for first-time users to determine their current location and navigate. In addition, visitors need to search for empty parking spaces, and after parking, it is difficult to remember the parking location (Some shopping malls install a camera at each parking location and provide the parking location by recognizing the license plate). In addition, visitors must find the stairs or elevators again to reach the destination in the building. Such situations cause discomfort to the user.

As the underground parking lot is the first indoor area that is entered from the outside, location tracking is necessary for a continuous navigation system. If positioning is possible in an underground parking lot, various services can be provided to users. When a vehicle enters an underground parking lot, the navigation screen should naturally switch from the outdoor map to the underground parking lot map. Further, it should guide the user to an empty parking space near the elevator closest to the destination. The parking location should be saved in the application and used as the destination when the user returns to the parking lot. In addition, when the user exits the vehicle, the pedestrian mode must be recognized and converted to an indoor navigation screen. When building a parking management center, based on the location result reported to the server by the application, the overall parking location of the vehicle can be identified in an underground parking lot. Thus, the users can be informed of the empty parking spaces, on entering underground parking lots. In large parking lots, there are several exits. If many vehicles are crowded at a certain exit, the navigation system can redirect the route to another exit.

Research related to positioning is generally divided into outdoor and indoor positioning. The global navigation satellite system (GNSS) [1] can provide stable location information outdoors, regardless of the location, time, and weather. However, because GNSS signals are not received in indoor spaces, the location must be estimated using another technology. To date, various studies have been conducted to estimate the locations in indoor spaces. Using pseudolites that transmit the same signals as the GNSS, the position can be estimated indoors [2,3]; however, in a wide coverage area, numerous pseudolites must be installed, and time synchronization between pseudolites is another problem. Although it is possible to estimate the position at a centimeter level using the ultra-wideband (UWB) [4], UWB transmitters must be installed indoors, similar to pseudolites. In addition, separate hardware is required to receive UWB signals. Bluetooth low energy (BLE) [5,6,7,8] is one of the most extensively used technologies in indoor location research, and the fingerprint method based on the received signal strength (RSS) or the angle of arrival (AOA) is available [9,10]. Research on indoor location estimation using vision [11] and light-emitting diodes (LEDs) [12] has also been conducted. Furthermore, pedestrian dead reckoning (PDR), which estimates the position of pedestrians using acceleration and gyroscopes, has been extensively studied [13,14,15,16]. This technology does not require the installation of additional infrastructure or a database (DB) for location estimation. However, because this technology accumulates using the step and heading information, the absolute position cannot be estimated, and errors accumulate over time. Therefore, the PDR technology has been investigated in combination with other RF signals. Moreover, research on indoor locations using WiFi signals has been actively performed [17,18,19]. In the case of WiFi signals, there is an advantage because numerous access points (APs) have already been installed in indoor spaces. However, when estimating the location using WiFi, a DB is required and must be managed well. WiFi APs can be easily installed or removed, and the location of the AP can be changed, which affects the WiFi positioning performance.

Although the need to estimate the location of vehicles in underground parking lots is increasing with the increase in the building area, the associated research has been limited. In [20], the location was estimated in an underground parking lot using dead reckoning (DR). A high-performance inertial navigation system (INS) is required to obtain high accuracy using DR. In addition, when DR alone is used, the position diverges over time; hence, it is necessary to combine it with other techniques for estimating the absolute position. Estimation of indoor locations using lighting infrastructure [21], vision [22], and wireless sensor networks such as Zigbee [23] has been investigated to estimate the position in an underground parking lot. In this study, we present an underground parking lot navigation system based on long-term evolution (LTE) signals [24]. The advantage of the LTE network is that the signal is very stable because it is managed by a telecommunication company. In the case of a Wi-Fi positioning system, the DB should be updated frequently because of the changes in the surrounding AP environment [25]. However, in the case of LTE, because it is not easy to install and remove the base station, signal repeatability in a specific area is better than WiFi signals. In addition, LTE signals can be received in all the areas with almost no shadow area. In an underground parking lot, only one or two LTE-based station signals are received. To estimate the accurate position using fingerprint technology, each specific position must have a unique signal pattern. Hence, accurate location performance can be realized only when as many AP signals as possible are received. However, in the case of LTE, because only one or two LTE base station signals are received, signal discrimination for each location is inferior. To solve this problem, this study utilizes an accumulated LTE signal sequence, i.e., the location is estimated using the LTE RSS for a certain period. In general, underground parking lots include several areas. When moving from a specific area to another area, various candidate groups from the DB are created, and the area with the pattern most similar to the user’s LTE signal pattern is estimated as the current location. Accordingly, in this study, extended subsequence dynamic time warping (ESDTW) is proposed for estimating the position of the vehicle. Within an area, it is possible to estimate the position using subsequence dynamic time warping (SDTW) [26]. Further, the rotation of the vehicle is determined through the gyroscope output to establish whether the vehicle is moving to a new area. In the new area, the location is estimated by comparing the new candidate trajectory with the user pattern using SDTW. The candidate generator generates new candidate trajectories using the estimated location and next link information. In addition, it is possible to determine the stopping and moving of a vehicle using the acceleration sensor of a smartphone. To confirm the performance of the proposed system, an experiment is conducted in the underground parking lot of a large shopping mall. The contributions of this study are as follows:

- The accurate position in an underground parking lot is estimated using the accumulation pattern of the LTE RSS. In particular, ESDTW is proposed to compare the LTE sequence and DB, and the effectiveness of the proposed technology is verified experimentally.
- The proposed system can estimate the position with sufficient accuracy for providing navigation services in an underground parking lot using only a mobile phone, without requiring additional infrastructure. This technology is easy to use, and it is possible to integrate it with the existing vehicle navigation system based on GNSS.

The remainder of this paper is organized as follows: Section 2 describes the configuration of the proposed system. Section 3 explains the proposed system and its algorithm. The experimental results and analysis are discussed in Section 4. Finally, the conclusions are presented in Section 5.

## 2. Block Diagram of the Proposed System and Its Applications

**Figure 1:**
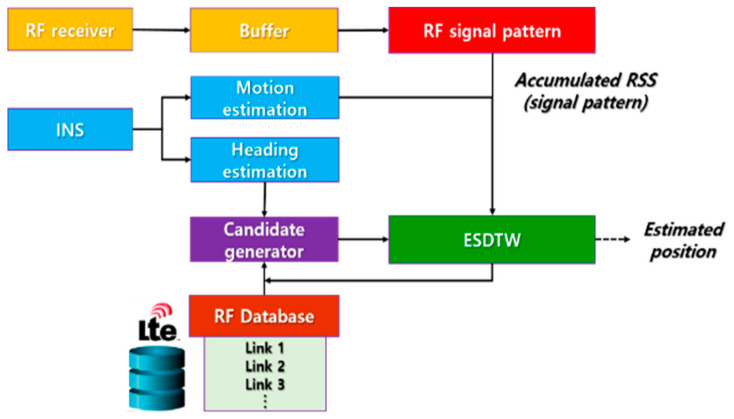
Block diagram of proposed system.

**Figure 2:**
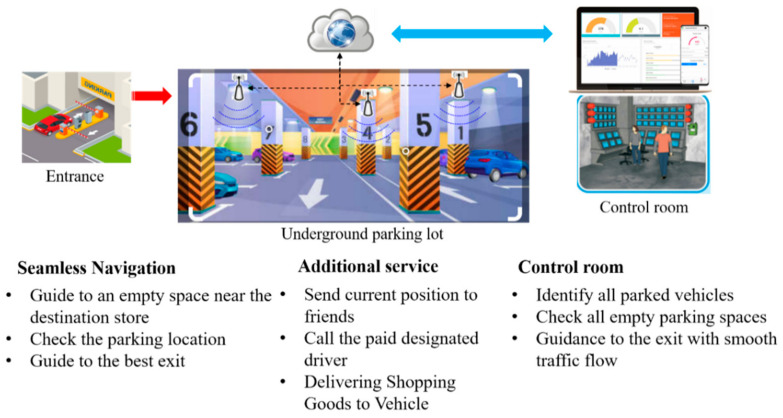
Overall proposed system configuration and its effectiveness.

Figure 1 shows the block diagram of the proposed system. A salient feature of the proposed system is the usage of an accumulated RF signal. Whenever the RF signal strength is received, it is stored in the user’s buffer. Using the acceleration sensor included in the mobile phone, the system checks the movement (stopped/moving) status of the vehicle. If it is determined that the vehicle has stopped, position estimation is not performed. The vehicle’s heading and turn motions are estimated using the gyroscope output. The candidate generator generates a candidate trajectory using the estimated position of the vehicle and the turn occurrence information. Finally, the vehicle’s position is estimated employing the proposed method, ESDTW, using the generated candidate trajectories and the user’s RF pattern. Figure 2 depicts the overall configuration of the proposed system and its effectiveness. The ultimate purpose of this research is to provide seamless navigation to users. As depicted in Figure 2, various services are possible using the proposed system, and other services can also be created in combination with the currently implemented service. Hence, a new location-based service (LBS) is offered to users.

## 3. Proposed Method

The proposed method is similar to the fingerprint, which is extensively used in indoor positioning systems. In the fingerprint method, it is necessary to create a DB by storing the RSS of the surrounding RF signals at each specific position defined in the testbed in advance. During the estimation phase of the actual position, the currently received signal pattern is compared with the DB, and the reference point (RP) with the RSS pattern most similar to the input RSS pattern is estimated as the present position of the user. However, this method significantly decreases the positioning accuracy when the number of RF beacons installed on the test bed is less or when the signal strength is weak. WiFi APs are not installed in an underground parking lot, and only LTE signals are received. In particular, because only one or two base station signals are received, accurate positioning cannot be performed with the existing fingerprint methods. To solve this problem, the accumulated RSS from the LTE was used. This following subsection describes the LTE DB construction, dynamic time warping (DTW), and the proposed method (ESDTW).

### 3.1. LTE DB Construction

#### Testbed

**Figure 3:**
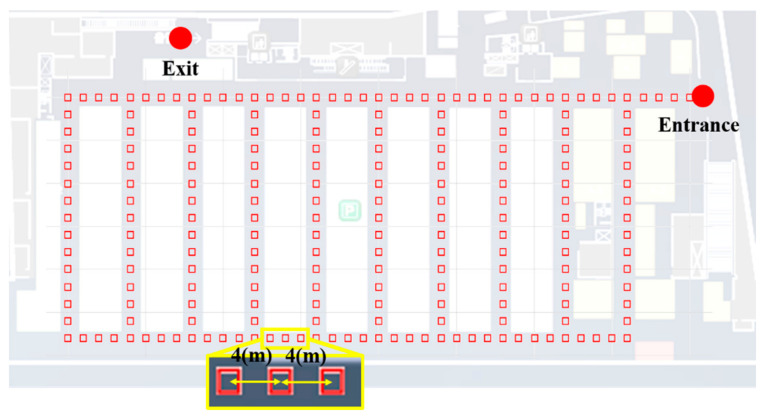
Reference points definition in underground parking lot.

**Figure 4:**
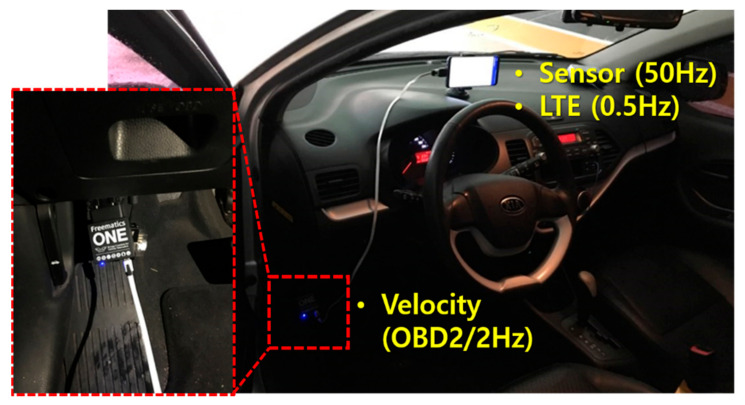
Experimental setting.

**Figure 5:**
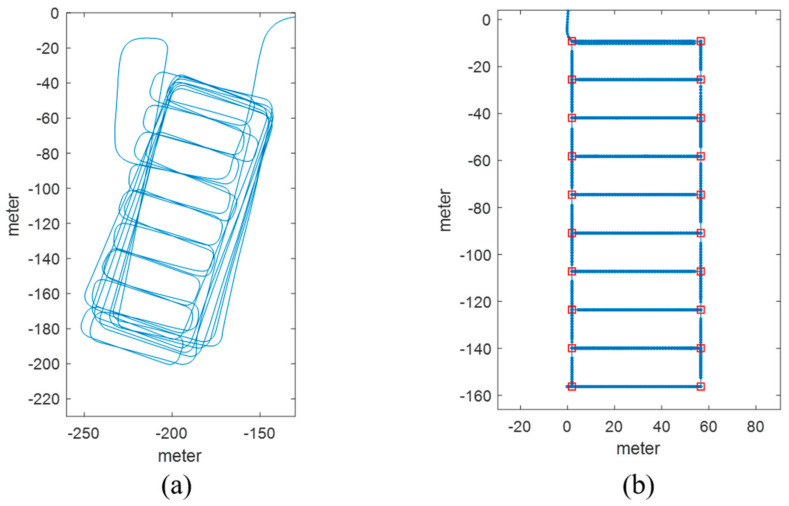
Vehicle trajectory by dead reckoning using OBD2 and smartphone. (**a**) Before map matching; (**b**) After map matching.

**Figure 6:**
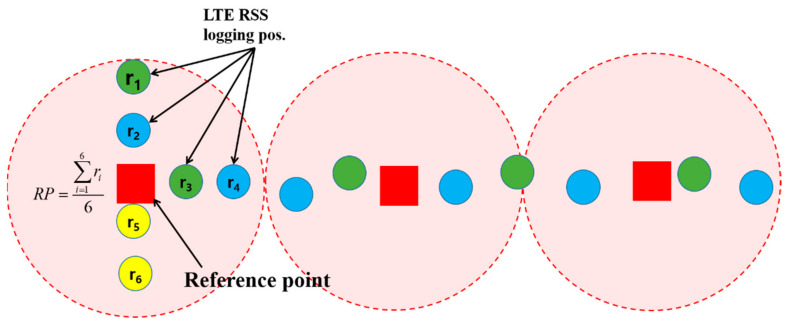
Calculation of RP value using LTE RSS collected data.

**Figure 7:**
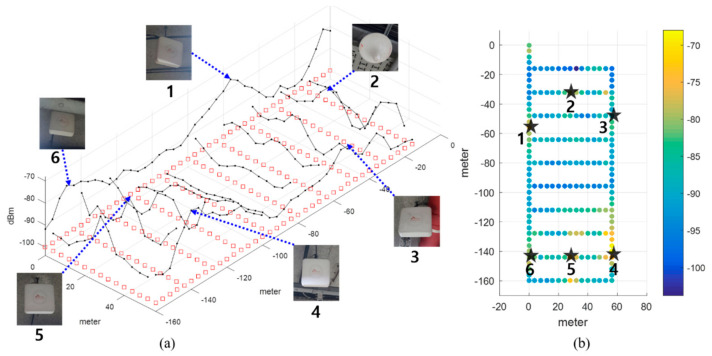
Overall RSS and RP of the LTE DB in the underground parking lot. (**a**) LTE RSS distribution of LTE DB; (**b**) Color map of LTE DB and LTE antenna positions.

In this study, an experiment was conducted in the underground parking lot of a large shopping mall. The parking lot involved a large area in which more than 500 vehicles could be parked. It was rectangular shaped and contained several corridors. To create the LTE DB, the RPs were configured at intervals of 4 m, as shown in Figure 3. The LTE DB also stores the LTE RSS information received from each RP. Figure 4 depicts the experimental settings. A smartphone was placed on the dashboard of the vehicle. The sensor data from the accelerometer and gyroscope and LTE signal were stored in the smartphone. To measure the distance traveled by the vehicle, velocity data were logged from the onboard diagnostic 2 (OBD2) and used as the reference data in the actual location estimation experiment as well as for creating the DB. The trajectory of the vehicle can be estimated using the logged velocity and direction information. Figure 5a displays the vehicle’s movement trajectory estimated using the OBD2 and gyroscope data. Figure 5b shows the map matching (MM) results. As the underground parking lot is in the form of a corridor, it can be defined as nodes and links. The red squares in Figure 6 indicate the nodes. When logging data for the LTE DB, the vehicle is turned only at a node. Therefore, when the turn of the vehicle is detected, the position of the vehicle is corrected at the node. As the vehicle moves, the accumulated error of the trajectory increases because of the velocity and heading errors; however, this accumulated error is corrected by performing MM at each node. Figure 6 illustrates the definition of the LTE signal strength at each RP. When the vehicle repeatedly moves along the same path, the LTE RSS sample points continue to increase around the RP. The value at the final RP is determined by averaging the LTE RSS sample points around the RP. LTE RSS samples of the same color have the same vehicle trajectory when passing through the corresponding RP. Figure 7 shows the overall RSS and RPs of the LTE DB in the underground parking lot. Figure 7a depicts the signal strength of the LTE signal received in 2D space. It can be observed that at a point where an LTE antenna is installed, the signal strength is high. It was confirmed that several antennas were installed in the underground parking lot. However, the physical cell identification (PCI) of the LTE signal received from each antenna was the same. Figure 7b shows the color map of the LTE DB signal strength. The closer the RP is to yellow, the stronger is the signal strength.

### 3.2. DTW

As the vehicle moves, the LTE RSS accumulates in the user buffer. The RP sequence that is most similar to the RSS sequence in the user buffer is the current vehicle location. DTW is an algorithm that determines the optimal alignment of two sequential data. Here, optimal alignment refers to index pairs with the minimum distance between two sequential data. The DTW finds a pair, even if the speed and length of the two time-sequences are different. We define two sequences *X* and *Y* as follows:

(1) $$X:=(x_{1},x_{2},x_{3},\;\ldots \;,x_{N}),$$

(2) $$Y:=(y_{1},y_{2},y_{3},\;\ldots \;,y_{M}),$$

where *N* and *M* are the lengths of sequences *X* and *Y*, respectively. The cost matrix *C* is defined as follows:

(3) $$C(i,j)=\left|x_{i}-y_{j}\right|\;1\leq i\leq N,1\leq j\leq M$$

The accumulated cost matrix *U_DTW_* is calculated as:

(4) $$\begin{array}{l} U_{DTW}(i,1)=\sum_{k=1}^{i}C(k,1)\\ U_{DTW}(1,j)=\sum_{k=1}^{j}C(1,k)\\ U_{DTW}(i,j)=\min\{U_{DTW}(i-1,j-1),U_{DTW}(i-1,j),U_{DTW}(i,j-1)\}+C(i,j) \end{array}$$

Finally, the optimal path *P* is obtained as

(5) P(i,j)=argmin{U(i−1,j−1),U(i−1,j),U(i,j−1)}

**Figure 8:**
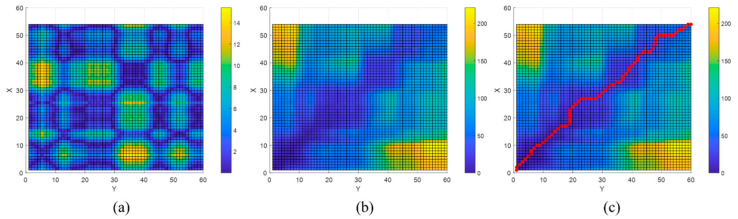
(**a**) Cost matrix; (**b**) Accumulated cost matrix; (**c**) Optimal path.

**Figure 9:**
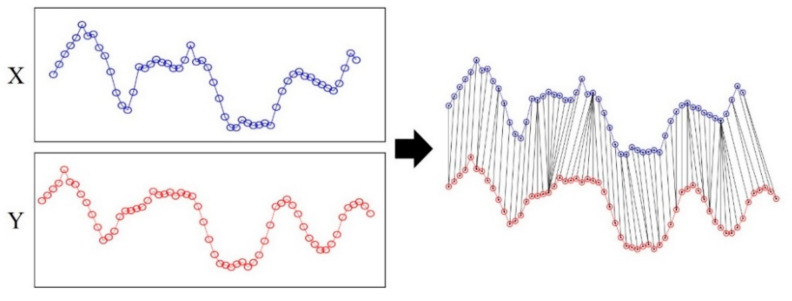
Two sequences connected by optimal path.

**Figure 10:**
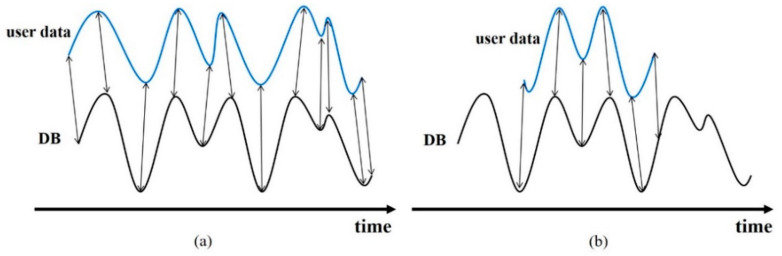
Comparison between DTW and SDTW. SDTW is applied when the physical length between sequences is different. (**a**) Aligned sequences using DTW; (**b**) Aligned sequences using SDTW.

Figure 8 shows an example of the described DTW. Suppose that the lengths of two sequences *X* and *Y* are 54 and 60. The sizes of *C* and *U* are 54 × 60, respectively. Figure 8a shows the cost matrix, whereas Figure 8b shows the accumulated cost matrix. Figure 8c displays the final selected optimal path P. Figure 9 depicts the two sequences *X* and *Y*. These two sequences are connected by an optimal path that includes the selected pairs. The DTW has the same start and end points as the *X* and *Y* sequences. However, there are cases where *X* is part of *Y*; i.e., if the physical sequence length of *X* is smaller than that of *Y*, the start or end point of *X* is the middle of the *Y* sequence. Figure 10 compares examples of DTW and SDTW. In this study, because the LTE RSS is increasingly accumulated, the position is estimated through SDTW. Algorithmically, DTW and SDTW are identical, except for one part; when calculating *U_SDTW_* in SDTW, the first row is obtained as follows:

(6) $$U_{SDTW}(i,1)=C(i,1)$$

This indicates that the first row-element of *U_SDTW_* is always initialized, and the minimum element of the last row of *U_SDTW_* is the last pair of optimal paths in SDTW.

### 3.3. Proposed Extended Subsequence DTW

**Figure 11:**
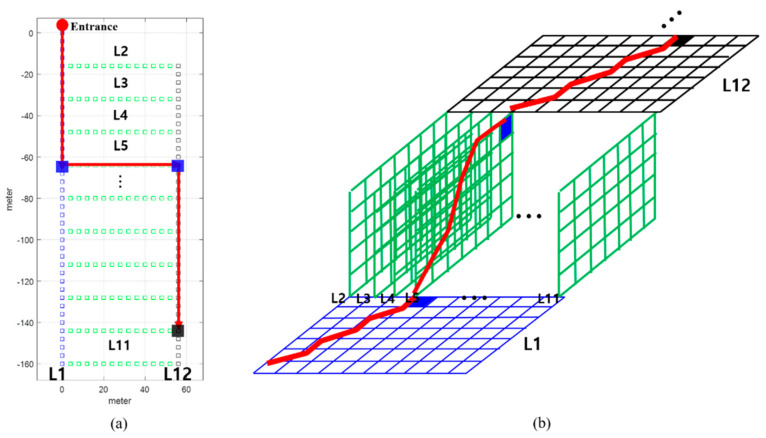
(**a**) Vehicle trajectory in the links; (**b**) Vehicle trajectory in the accumulated cost matrix.

**Figure 12:**
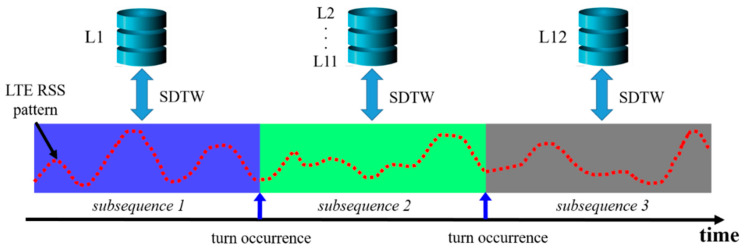
ESDTW is executed using the user buffer and candidate DB. The user buffer is divided into subsequences based on the vehicle’s turn motion. Each SDTW is performed using the subsequence and selected link.

In this subsection, the ESDTW proposed in this study is described. An underground parking lot includes several links. As the vehicle moves, it passes through a specific link. As previously mentioned, the LTE RSS accumulates in the user buffer as the vehicle moves and the location of the vehicle in a specific link is estimated through SDTW. When the vehicle enters the next link, the location is estimated using the user buffer and the link combination, in which two links are connected. Here, we call the link combination as the candidate trajectory, which is a combination of links through which the vehicle can move. Moreover, the candidate trajectories are continuously expanded with the addition of new links. The candidate trajectories are generated by the candidate generator using the motion information of the vehicle and the previously estimated position. The candidate trajectory containing the links is the candidate for the current location of the vehicle. Figure 11 shows the accumulated cost matrix of the ESDTW according to the vehicle’s movement trajectory. In Figure 11a, there is a total of 12 links, including two vertical and ten horizontal links. The red line denotes the movement trajectory of the vehicle. The blue square denotes the location at which a turn occurs. When a vehicle turns, the candidate trajectory is expanded again through the links that exist after the turn. The black square denotes the vehicle’s final position. In Figure 11b, the red line denotes the final optimal path. As the current vehicle is on link 12, there is a vehicle in the accumulated cost matrix of link 12. If the vehicle enters the green link again, the accumulated cost matrix continues to expand. Figure 12 depicts the situation displayed in Figure 11. The user buffer is divided by the turning motion of the vehicle. The candidate generator generates a possible candidate trajectory containing the accessible links. After the divided buffer subsequence is compared with the link in each candidate DB, the location of the vehicle is determined through the candidate trajectory with the least cost. This method minimizes the candidate trajectory with which the user buffer is compared. This reduces the computational complexity and positional errors.

## 4. Experimental Results

### 4.1. Testbed and Experimental Setup

**Figure 13:**
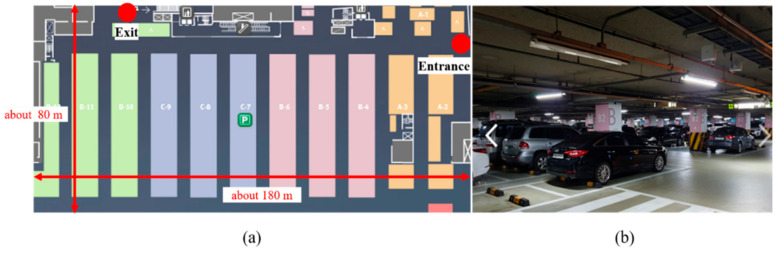
(**a**) Map of testbed; (**b**) Front view of underground parking lot.

To validate the proposed method, an experiment was conducted in the underground parking lot of a super supermarket (SSM) in Jayang-dong, Seoul. Figure 13 shows the corresponding test bed. LTE signals were received in all the areas of the underground parking lot. There were no radio shadow areas, but a very weak signal was received in a particular place, which was far from the LTE antenna.

**Figure 14:**
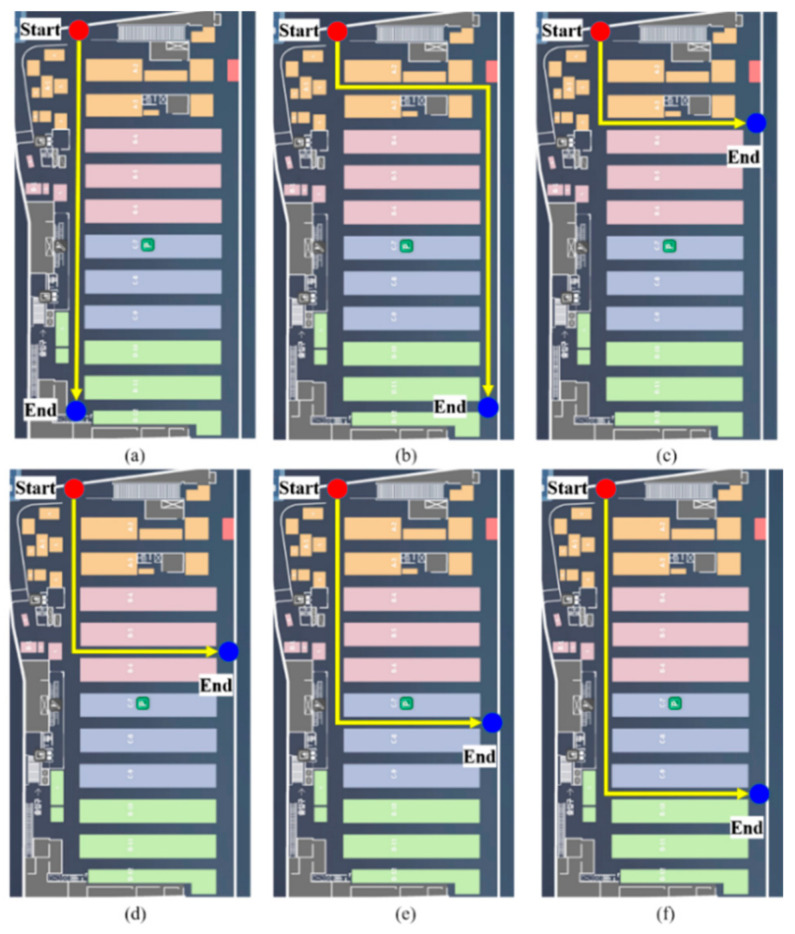
Test scenarios. (**a**) scenario 1; (**b**) scenario 2; (**c**) scenario 3; (**d**) scenario 4; (**e**) scenario 5; (**f**) scenario 6.

Figure 4 depicts the experimental settings. The experiment was conducted by placing a smartphone on the dashboard. The sensor data, LTE RSS, and OBD2 data were stored in the smartphone. Along with each saved data, the internal clock time was saved, and each data was synchronized using this time. The performance of the proposed method was analyzed by post-processing using the stored data. As mentioned in Chapter 3, velocity data from the vehicle’s OBD2 were used as the reference data to evaluate the performance of the proposed system. The distance moved by the vehicle was estimated using the velocity data of OBD2. The heading was estimated using the gyroscope output, and the reference trajectory was estimated by performing MM. The accumulated error was eliminated by detecting the time at which the vehicle turned and matching the position to a nearby corner point. The mobile phone used in the experiment was the Samsung Galaxy 9+. The OBD2 and sensor data were logged at approximately 2 Hz and 50 Hz, respectively. The LTE RSS was updated every 2 s. Figure 14 illustrates the experimental scenarios. A total of six experiments was conducted. Scenarios 1 and 2 evaluated the performance in a straight line; whereas scenarios 3–6 checked the accuracy of position estimation when entering a new link.

### 4.2. Result Analysis

**Figure 15:**
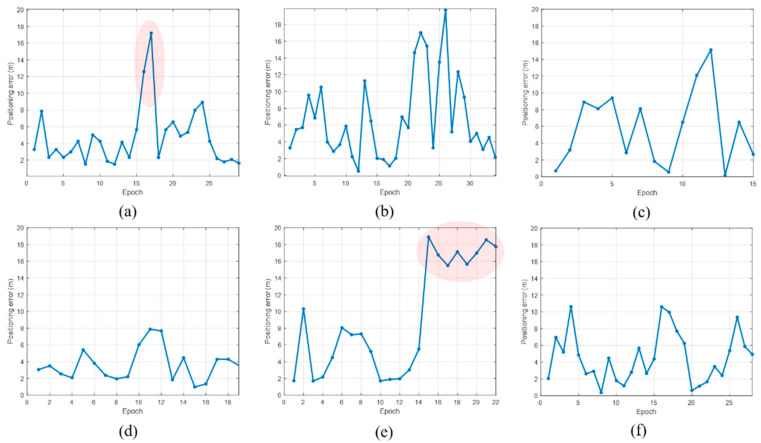
Positioning error by epoch for each scenario. (**a**) position error of scenario 1; (**b**) position error of scenario 2; (**c**) position error of scenario 3; (**d**) position error of scenario 4; (**e**) position error of scenario 5; (**f**) position error of scenario 6.

**Figure 16:**
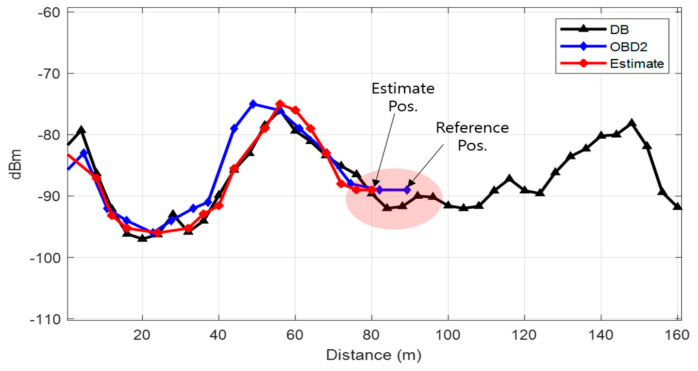
The epoch with the biggest error in scenario 1. Weak RSS is continuously received in this area.

**Figure 17:**
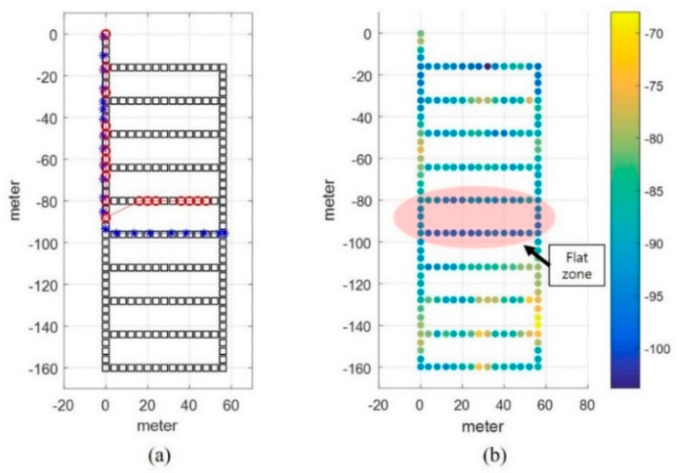
(**a**) 2D estimated position in scenario 5; (**b**) RSS distribution in L5 and L6.

**Figure 18:**
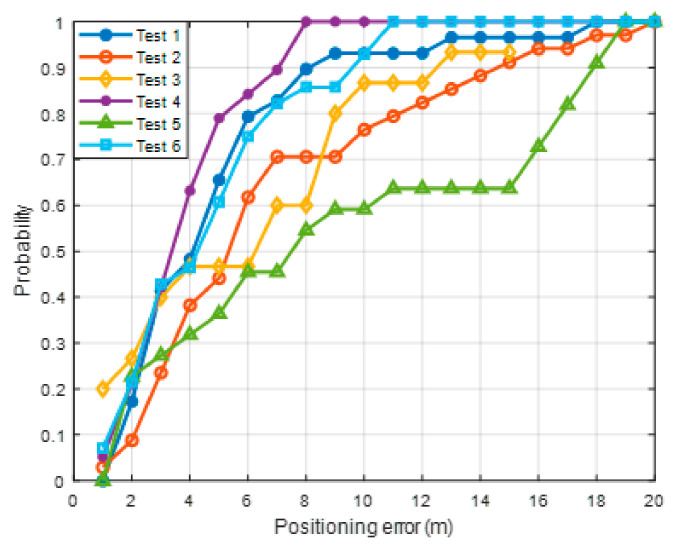
Cumulative distribution function (CDF) for all scenarios.

Figure 15 displays the positioning results for all the scenarios. Overall, the level of error is within 20 m, but the positioning results differ for each scenario. In particular, the red circle indicates the part where the positioning error is large. Due to the nature of the proposed method, location accuracy tends to be low in regions where RPs with weak signal strength are uniformly distributed. To accurately estimate the user position with DTW, it is advantageous to show unique RSS characteristics for each RP. Figure 16 compares the user buffer and DB candidate when the positioning error is maximum in scenario 1. Both the DB candidate and the received LTE RSS are weak in the epoch with large positioning error. If a weak signal is continuously received, there is a possibility that the position error may increase in an underground parking lot environment where only one LTE PCI is allocated. Figure 17a shows the 2D position results for scenario 5. The vehicle moves to link C6, but its position is estimated using link C5. The LTE RSS in the region is also weak, and because the LTE signal noise is greater than the difference in the signal strength between the two links, the position is incorrectly estimated. Figure 18 presents the cumulative distribution function (CDF) of the positioning errors for each scenario. Scenarios 4 and 6 show highly accurate location-estimation results. Scenarios 2 and 5, which pass through the weak signal section, show reduced location estimation performance compared to the other scenarios. Table 1 summarizes the positioning errors for all the test scenarios; the average error is less than 10 m for all the scenarios. The crucial aspect to consider in this study is the maximum error. To perform user-oriented and stable navigation, it is necessary to reduce the maximum error. The maximum error is less than 20 m in all the scenarios.

### 4.3. Discussion

The LTE signal-based underground parking-lot location estimation system has the advantage of estimating the location using a smartphone alone, without requiring any additional infrastructure for location estimation. There is currently no alternative for estimating the location of a vehicle in an underground parking lot, and the proposed method offers a new navigation system. However, because of the characteristics of the LTE network, only one LTE PCI is allocated and serviced in an underground parking lot. In addition, although LTE signals reach all the areas in an underground parking lot, there are many areas where the signal strength is weak; hence, the location error tends to decrease in these areas. This problem can be solved by installing a BLE beacon in the area where a weak signal is received. As BLE signals can also be received by smartphones, it is possible to compensate for the areas where the LTE RSS is weak. However, the cost of maintenance and management of the additional infrastructure must be considered. Another method is to estimate the moving distance of the vehicle using an acceleration sensor. When using the navigation application, because the smartphone is fixed to the dashboard, it is possible to obtain relatively stable acceleration sensor output values. If attitude estimation of the smartphone and calibration can be performed accurately, the use of an acceleration sensor may be an option.

## 5. Conclusions

As the services of vehicle navigation applications do not extend to underground parking lots, a user entering the parking lot must drive the vehicle entirely depending on his/her own vision. It is not easy for a user to find an appropriate empty parking space in a spacious and complex underground parking lot. Moreover, it is difficult to find an escalator or elevator close to the destination, after parking. To solve this problem, an underground parking lot navigation system using an accumulated LTE signal was presented in this study. The advantage of the proposed system is that it is possible to navigate using a smartphone alone, without installing additional infrastructure. The candidate trajectory is generated by a DB candidate generator, and the vehicle position is estimated by comparing the user buffer data and candidate trajectory through ESDTW. To evaluate the effectiveness of the proposed system, a field test was conducted and accurate positioning results were obtained.

In the future work, we will improve the location estimation performance by combining the proposed technology and dead reckoning technology and then use this system in multi-storey underground parking lots. As the proposed technology is combined with the existing navigation system, users do not need to install a separate application. When a vehicle enters the underground parking lot, the navigation screen automatically changes to the underground parking lot map, allowing a driver to experience a new seamless navigation.

## Figures and Tables

**Table 1:** Analysis of ESDTW positioning error.

Test Num.	Mean (m)	RMSE (m)	Max (m)	CEP (m)
1	4.68	5.87	17.19	4.13
2	6.68	8.32	19.73	5.32
3	5.78	7.35	15.14	6.50
4	3.64	4.15	7.87	3.50
5	9.06	11.26	18.89	7.27
6	4.56	5.47	10.62	4.43

## Data Availability

Not applicable.
